# The Use of the QbD Approach to Optimize the Co-Loading of Simvastatin and Doxorubicin in Liposomes for a Synergistic Anticancer Effect

**DOI:** 10.3390/ph15101211

**Published:** 2022-09-29

**Authors:** Cristina-Ioana Barbalata, Alina Silvia Porfire, Tibor Casian, Dana Muntean, Iulia Rus, Mihaela Tertis, Cecilia Cristea, Anca Pop, Julien Cherfan, Felicia Loghin, Ioan Tomuta

**Affiliations:** 1 Department of Pharmaceutical Technology and Biopharmaceutics, Faculty of Pharmacy, “Iuliu Hatieganu” University of Medicine and Pharmacy, 41 Victor Babes Street, 400012 Cluj-Napoca, Romania; 2 Department of Analytical Chemistry and Instrumental Analysis, Faculty of Pharmacy, “Iuliu Hatieganu” University of Medicine and Pharmacy, 4 Louis Pasteur Street, 400349 Cluj-Napoca, Romania; 3 Department of Toxicology, Faculty of Pharmacy, “Iuliu Hatieganu” University of Medicine and Pharmacy, 6 Louis Pasteur Street, 400349 Cluj-Napoca, Romania; 4 BCBS Team (Biotechnologies et Chimie des Bioressources Pour la Santé), LIENSs Laboratory (Littoral Environment et Sociétés), UMR CNRS 7266, University of La Rochelle, 17000 La Rochelle, France

**Keywords:** QbD, liposomes, chronoamperometry, synergism

## Abstract

The present study aimed to optimize a liposomal formulation co-encapsulating simvastatin (SIM) and doxorubicin (DOX) that has future perspectives in anticancer therapy. The optimization process was performed by implementing the Quality by Design concept and by considering the results of a previous screening study. Failure Mode and Effects Analysis was used for the identification of the potential critical factors, i.e., phospholipid, SIM and DOX concentration, which were assessed in an optimization experimental design with the purpose of designing an optimal formulation. The optimal formulation, meeting the established quality profile, was additionally characterized in terms of the release profile and antiproliferative effects. During dissolution studies, a novel chronoamperometric method was used for the simultaneous quantification of SIM and DOX. The obtained data confirmed the similarity of this method with a validated HPLC method. The anticancer potential of the optimal formulation was tested against two human cancerous cell lines, namely T47D-KBluc human mammary ductal carcinoma cell line and A549 human pulmonary cancer cell line. The results highlighted that the antiproliferative effect of the optimal formulation is concentration dependent and favors a synergistic effect of the two drugs.

## 1. Introduction

The increased number of cancer cases and the negative predictions of cancer mortality are urging the scientific community to develop a better understanding of cancer biology and to develop new treatment options [1]. One of the factors responsible for cancer drug resistance and cancer poor prognosis is cholesterol. The latest evidence highlighted that cancer cells are able to store high levels of cholesterol in a free or esterified form, influencing cellular membrane properties such as fluidity or permeability. As a consequence, the infiltration of chemotherapeutic drug molecules inside the cancerous cells is hampered, leading to drug resistance and reduced anticancer therapeutic response. Moreover, it has been proved that certain cancer cell lines, i.e., prostate, gastric or colon cancer cell lines, are able to synthetize cholesterol de novo due to an overexpression of 3-hydroxy-3-methyl-glutaryl-coenzyme A (HMGCoA) reductase, a rate limiting enzyme in the synthesis of cholesterol [2,3,4].

At present, the anticancer therapies are varied, from classical chemotherapeutic drugs to immunotherapy [5]. Nevertheless, the most preferred anticancer treatment option remains the administration of chemotherapeutic drugs with an increased risk of adverse reactions that result in reduced patient compliance and supplementary healthcare costs [6]. One such example is doxorubicin (DOX), which is used in the treatment of various types of tumors, such as breast tumors, lung tumors or ovarian carcinomas [7], but it also induces multi organ toxicity, particularly cardiotoxicity [8]. On the other hand, immunotherapy is usually high priced, causing hesitations or interruptions in following the therapeutic scheme [5]. The latest studies have proposed the repurposing of non-cancer drug molecules, i.e., antidepressants, antifungals, antidiabetics, statins or cardiovascular drugs, to reduce the time and costs of developing new anticancer molecules [9]. Among these, statins were proved to be beneficial in cancer prognosis due to their ability to inhibit the activity of HMGCoA reductase, thus preventing the synthesis of cholesterol and its derivatives [2].

To avoid drug resistance, anticancer therapeutic schemes are recommending the use of two or more chemotherapeutic drugs with synergistic effect to target multiple intracellular pathways, which will ultimately lead to the inhibition of tumor progression [6,10]. In this respect, the latest evidence highlighted great interest regarding the association of statins with various chemotherapeutic drugs in the treatment of breast and lung cancer [11,12]. Even though SIM in combination with DOX has not been investigated yet in clinical trials for a synergistic anticancer effect, their association presented a synergistic effect on numerous cancer cell lines such as breast or colon cancer cell lines [13,14]. Despite this, the low therapeutic index, the non-specific targeting and different pharmacokinetic profiles of the drugs are raising difficulties in achieving the desired therapeutic outcome. To overcome these barriers, liposomes represent a promising option in the delivery of two or more active substances at the tumor site due to their ability to incorporate drugs with different physiochemical properties [15]. On the other hand, the synergistic or additive effect between two active substances was evidenced to be dependent on the incorporated drug ratio or drug release profile of liposomes [15].

Drug development studies usually involve the use of large amounts of financial resources and time [16]. For this reason, the implementation Quality by Design (QbD) approach in drug product development has gained considerable interest in both academia and pharmaceutical industry [16,17]. The benefits that the QbD approach brings in pharmaceutical development are related to the in-depth understanding of the manufacturing process within a short period of time and with minimal costs [18,19]. The use of the QbD approach in the development of nanopharmaceutical formulations is encouraged due to the complex formulations and preparation processes while maintaining the quality attributes within limits to achieve the quality target product profile (QTPP) [20].

Considering these, this study aimed to optimize a liposomal formulation co-encapsulating SIM and DOX using the QbD approach, and to perform a complete in vitro characterization of the optimal formulation. The optimization study took into account the results we obtained in the screening study of the same liposomal formulation [14]. More precisely, in the previous study, we screened three formulation factors and two process parameters to emphasize the manner in which these factors influence the quality attributes of liposomes, i.e., size, polydispersity index (PdI), zeta potential, liposomal morphology, drug release profile and the antiproliferative potential of SIM and DOX combination. The formulation factors that were previously evidenced to have a significant impact on the quality attributes of liposomes, namely the phospholipid (PL) concentration, SIM and DOX concentration, were optimized in this study using the design of experiments (DoE), the final goal being the achievement of an optimal formulation that complies with the QTPP. The in vitro characterization of the optimal formulation consisted of characterizing the drug release profile using two different analytical techniques (HPLC and chronoamperometry), performing the stability study, and assessing the cytotoxic profile on two human cancer cell lines, namely T47D-KBluc human mammary ductal carcinoma cell line and A549 human pulmonary cancer cell line. Compared to the previous study [14], this study intends to highlight that drug repurposing can be a valuable instrument in the fight against cancer by assessing the same drug combination in a free or liposomal form against two different human cancer cell lines, and that drug concentration is not the only factor that influences the anticancer potential of SIM and DOX combination, but also the biology of the cancerous cell line. Basically, the novelty of this study is founded on three sections: (1) the use of the QbD approach in the optimization process of a liposomal formulation co-encapsulating SIM and DOX; (2) the use of an innovative analytical technique to simultaneously quantify SIM and DOX from the release medium; and (3) highlighting the differences in the antiproliferative effects of the same liposomal formulation on two different human cancer cell lines.

## 2. Results and Discussions

### 2.1. The Optimization Process of SIM-DOX-LCL

Usually, the implementation of the QbD approach starts by defining the QTPP and ends with establishing the control strategy [18]. Setting the QTPP in the first steps of the development process helps the research group to focus on designing a product that meets both the patient needs and the international regulation [21]. The QTPP generally contains features about the formulation, route of administration, release profile and other quality attributes such as morphology, crystallinity, water content, etc. [18]. In view of this, the current study aimed to optimize a former developed liposomal formulation co-encapsulating SIM and DOX that has perspectives in cancer therapy [14]. To accomplish this target, the long circulating liposomes loaded with SIM and DOX (SIM-DOX-LCL) were designed for intravenous administration to increase the bioavailability of both SIM and DOX due to their intensive hepatic metabolization [22,23]. Moreover, the choice of a liposomal formulation can reduce the intensity and the number of systemic side effects caused by the high doses of SIM and DOX needed in cancer therapy [8,24,25].

According to a World Health Organization report, the first three most prevalent types of cancer in 2020 were breast, lung and colon and rectum cancer, accounting for more than three million deaths worldwide [26]. This fact emphasizes the need to discover new drugs for cancer treatment, particularly for these three types of cancer. As a continuation of our previous study, which demonstrated that SIM can enhance the antiproliferative properties of DOX on C26 murine colon cancer cell line co-cultured with macrophages [14], in the present study we intended to evaluate the same drug combination as free form and co-encapsulated in liposomes against two human cancerous cell lines, namely T47D-KBluc human mammary ductal carcinoma cell line and A549 human pulmonary cancer cell line. The benefits of associating statins with chemotherapeutic drugs have been demonstrated in both preclinical and clinical studies. The results have evidenced that an anticancer effect can be obtained in cancers such as lung cancer, breast cancer or prostate cancer [27]. Moreover, the idea of repurposing SIM is in line with the latest trends of the European Medicine Agency, which started a pilot project aiming at drug repurposing to provide new therapeutic alternatives in conditions where the actual therapy is associated with numerous disadvantages, such as multi-organ side effects [28]. On the other hand, a thorough understanding of cancer biology is essential for the selection of the appropriate drug combinations and administered doses, to achieve a synergistic effect in anticancer treatment [29]. By assessing the same drug combination on two different human cancer cell lines using identical concentration levels, the differences in the antiproliferative responses will be determined by the differences in cellular biology, thus providing a better understanding of the influences that affect the anticancer activity of SIM and DOX combination.

Yet, a liposomal formulation can be considered suitable for in vivo administration only if the quality attributes of liposomes, i.e., size, PdI, zeta potential, are attaining the target values established by the scientific community. More precisely, the liposomes should be in the size range of 50 to 200 nm and monodispersed (PdI < 0.2) to guarantee the permeation and retention of the nanoparticles in the tumor, and to avoid their uptake by the macrophages [30]. On the other hand, a zeta potential value lesser than −20 mV is preferred because it secures long-term stability and the developed nanoparticles are not noxious to health cells or blood components [31]. In addition, we considered it essential that the drug release profile of both SIM and DOX be extended in order to avoid early drug release in the blood [32], which can further lead to the occurrence of systemic side effects and the failure to ensure the right drug ratio at the tumor site.

**Figure 1:**
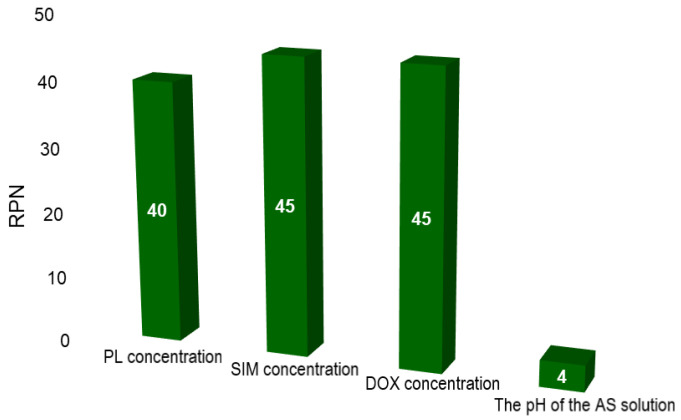
The RPN of each factor evaluated via FMEA.

The next step in the QbD approach is performing risk assessment, which has the role of identifying and quantifying the potential risk that endangers the achievement of the QTPP [18]. According to the International Council for Harmonization of Technical Requirements for Pharmaceuticals for Human Use (ICH) guideline, risk assessment “is typically performed early in the pharmaceutical development process and is repeated as more information becomes available and greater knowledge is obtained” [18]. In the previous work [14], risk identification via an Ishikawa diagram, and risk evaluation via Failure Mode and Effects Analysis (FMEA) were performed to identify those factors that might have a critical impact on the critical quality attributes (CQAs) of SIM-DOX-LCL formulation. Three formulation factors, namely the PL concentration, SIM and DOX concentration, and two process parameters, namely the pH of the ammonium sulfate (AS) solution and the incubation time of long circulating liposomes loaded with SIM (SIM-LCL) with DOX were formerly identified as potentially critical and were studied in a screening experimental design [14].The obtained results were used in the current study as an outset for the optimization of SIM-DOX-LCL formulation. As the screening study has pointed out, PL, SIM and DOX concentrations, as well as the pH of the AS solution, presented the greatest influence on SIM-DOX-LCL CQAs [14]. Therefore, these factors were re-evaluated via FMEA and the scores are plotted in Figure 1.

Compared to the previous results [14], the risk priority number (RPN) for all factors decreased (Figure 1). This was possible through the screening study, which provided an overview of the influences that the studied factors have on the CQAs of the SIM-DOX-LCL formulation, and contributed to the understanding of these effects. Nevertheless, the PL, SIM and DOX concentration still have the highest RPN. The criticality of these three factors arises from their major role in assuring the right ratio between SIM and DOX in the final formulation, as well as in guaranteeing a high encapsulation efficiency (EE%) [14,33]. The previous work demonstrated that the use of a low concentration of PL results in low EE% and entrapped drug concentration [14] and therefore, higher levels of PL were proposed for the optimization DoE (between 44 and 77 mM). On the other hand, the pH of the AS solution became non-critical. The reason for this is that DOX can achieve a higher EE% when the pH of the AS solution is five [14] and consequently, the central issue becomes the control method of the pH.

Considering these results, the concentrations of PL, SIM and DOX were studied as independent variables in an optimization DoE, while the entrapped drug concentration, EE%, liposomal size, PdI, and zeta potential were the dependent variables. The composition of the formulations and the experimental results of the DoE are presented in Table 1.

The experimental results were fitted through multilinear regression model and the statistical parameters of the fitting model and Analysis of Variance (ANOVA) test are presented in Table 2.

The statistical parameters of the summary of fit evidenced that the results fit well with the proposed model. However, the ANOVA statistical parameters showed that the formulation factors had a great impact on the responses, and there is no lack of fit for either of the responses (*p* > 0.1).

The equation that describes the influences of the formulation factors on the responses is:

Y_n_ = b_0_ + b_1_X_1_ + b_2_X_2_ + b_3_X_3_ + b_11_X_1_^2^ + b_22_X_2_^2^ + b_33_X_3_^3^ + b_12_X_1_X_2_ + b_13_X_1_X_3_ + b_23_X_2_X_3_ (1)

where Y_n_ is the response in question, b_0_ is the model constant, b_1_, b_2_ and b_3_ are the linear coefficients, b_11_, b_22_ and b_33_ are the quadratic coefficients, and b_12_, b_13_ and b_23_ are the interaction coefficients of the three factors.

**Figure 2:**
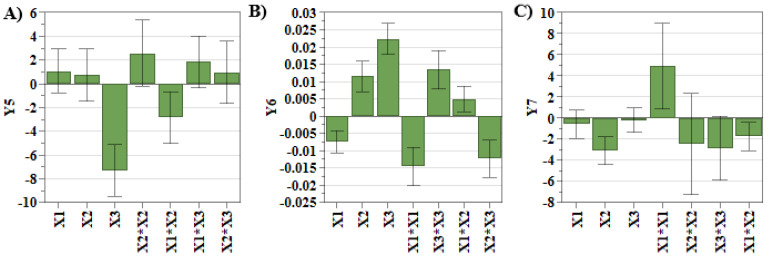
Coefficient plots for liposomal size (**A**), PdI (**B**) and zeta potential (**C**). **X_1_**: PL concentration (mM); **X_2_**: DOX concentration (mM); **X_3_**: SIM concentration (mM); **Y_5_**: Liposomal size (nm); **Y_6_**: Liposomal PdI; **Y_7_**: Zeta potential (mV).

By looking at the experimental results (Table 1), it can be observed that the liposomes dimension was in the size range of 105.5 nm and 169.4 nm, the PdI was between 0.02 and 0.08, and zeta potential values were below −20 mV. These results highlight that the referred quality attributes are within the established target ranges, and no additional optimization is required. However, the coefficients that show the manner in which each factor influenced the studied responses are presented in Table 3 and Figure 2.

Figure 2A,B evidence that SIM concentration had the greatest influence on the liposomal size and PdI. As shown in our previous work, SIM presents a stiffening effect on the lipid bilayer, causing ruptures in the liposomal membrane during the extrusion process. Due to this, the newly formed liposomes are small in size and polydisperse [14,34,35]. Another factor that causes the increase in PdI values is DOX concentration (Figure 2B). During the incubation step of SIM-LCL with DOX, DOX crosses the lipid bilayer due to a pH gradient and precipitates inside the liposomes as DOX sulfate. When high concentrations of DOX are used, the size and the shape of DOX sulfate crystals vary, influencing the shape of liposomes and the PdI values [14,36]. On the contrary, the use of high concentrations of PL results in the formation of a greater number of liposomes that can encapsulate DOX, and consequently, the dimensions of DOX sulfate crystals decrease [14]. In addition to these, DOX concentration also has a significant impact on zeta potential response. More specifically, by increasing the concentration of DOX, the zeta potential values decrease (Figure 2C). During the incubation step of SIM-LCL with DOX, DOX hydrochloride dissociates outside the liposomes in DOX, chloride ions and protons. DOX in a neutral form crosses the lipid bilayer, where it precipitates as DOX sulfate, and the resulting ammonium molecules from the dissociation of AS diffuse outside the liposomes [37]. Considering these, we assume that the chloride ions resulted from the dissociation of DOX chloride exceed that of ammonia, and therefore, the zeta potential decreases with the increase in DOX concentration.

**Figure 3:**
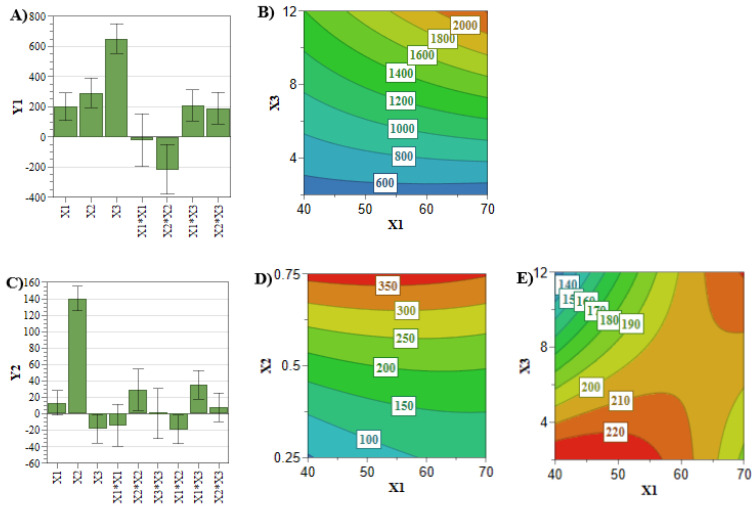
Coefficient plot for SIM (**A**) and DOX (**C**) entrapped concentration. Response contour plot for SIM entrapped concentration (**B**) and DOX entrapped concentration (**D**,**E**). **X_1_**: PL concentration (mM); **X_2_**: DOX concentration (mM); **X_3_**: SIM concentration (mM); **Y_1_**: SIM entrapped concentration (µg/mL); **Y_2_**: DOX entrapped concentration (µg/mL).

**Figure 4:**
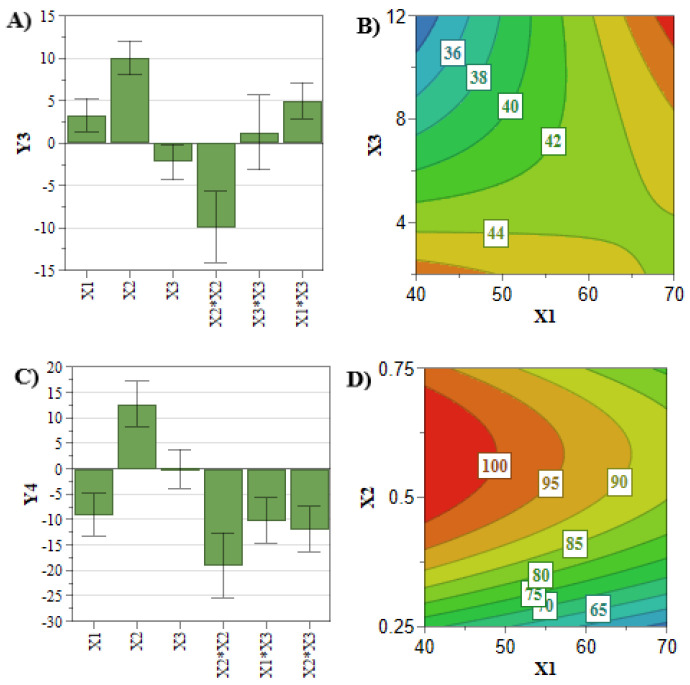
Coefficient plots for SIM (**A**) and DOX (**C**) EE%. Response contour plots for SIM (**B**) and DOX (**D**) EE%. **X_1_**: PL concentration (mM); **X_2_**: DOX concentration (mM); **X_3_**: SIM concentration (mM); **Y_3_**: SIM EE% (%); **Y_4_**: DOX EE% (%).

In comparison with the previously analyzed quality attributes, the entrapped drug concentration and the EE% responses did not attain the target values for all the experiments of the DoE. Therefore, an in-depth analysis of these two CQAs is mandatory for the optimization process. Achieving a high entrapped drug concentration and EE% is desirous in terms of costs and administered dose [33]. These two responses are mainly influenced by the concentration of the PL and active pharmaceutical ingredient (API) (Figure 3 and Figure 4) [33]. In this respect, Figure 4A,B highlighted that by increasing the PL and SIM concentrations, the SIM EE% increases due to an increase in the number of formed liposomes that can encapsulate more API [38]. On the other hand, Figure 3A–D show that the API concentration had the greatest influence on the entrapped drug concentration of SIM and DOX, and these responses increased with the use of elevated concentrations of SIM or DOX.

However, when co-encapsulating two APIs with different physiochemical properties, interferences in the encapsulation process of the APIs might occur [39,40]. In this view, the interaction coefficients (Table 3) demonstrated linkage between SIM and PL concentrations for DOX entrapped concentration plotted in Figure 3E. It can be noted that the highest DOX entrapped concentration was achieved at low levels of PL and SIM concentration, and inversely. This might be the consequence of the lipophilic character of SIM, which could prevent DOX from crossing the liposomal membrane during the incubation process, leading to a reduced entrapped concentration [40]. At the same time, using high levels of SIM and PL hinders the extrusion process, leading to a reduction in SIM entrapped concentration and lipophilic character of liposomal membrane, thus promoting the encapsulation of DOX [14].

These mathematical relationships established between input and output provide the scientific basis for defining the design space for SIM-DOX-LCL formulation [18,21]. The design space and the optimal formulation for SIM-DOX-LCL were generated using the optimization function of Modde 13 software after establishing the target ranges for each response.

**Figure 5:**
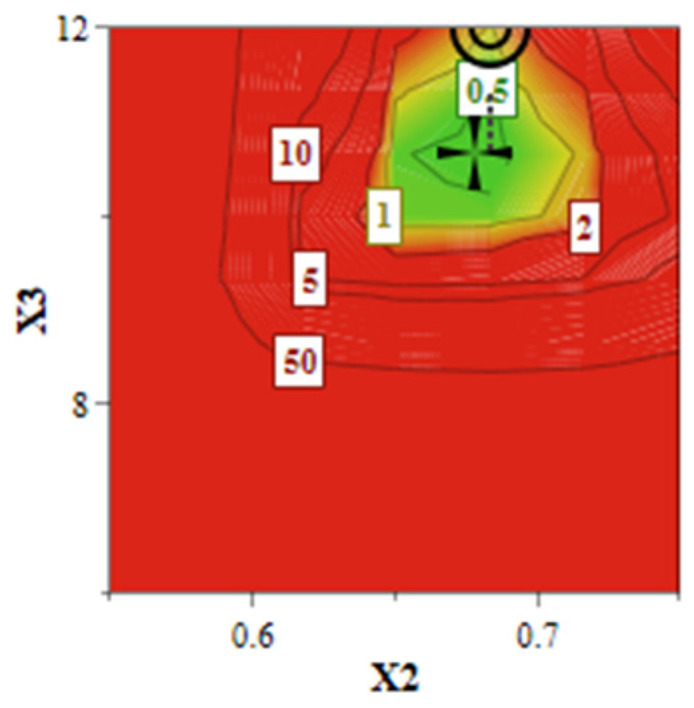
The Design Space of SIM-DOX-LCL formulation. X_2_: DOX concentration (mM); X_3_: SIM concentration (mM).

As illustrated in Figure 5, the design space (green area) suggests that the achievement of a liposomal formulation that meets the QTPP is conditioned by the use of increased levels of SIM and DOX, attesting to the criticality of these two factors. The quantitative formulation of the optimized SIM-DOX-LCL is presented in Table 4 along with the predicted values and the experimental results, showing that these two values are very close. In other words, it can be said that SIM-DOX-LCL optimal formulation complies with the QTPP, since the experimental results meet the target ranges.

### 2.2. In Vitro Release Study

#### 2.2.1. The Evaluation of SIM and DOX Release Profile from the Optimal Formulation Using the HPLC Method

**Figure 6:**
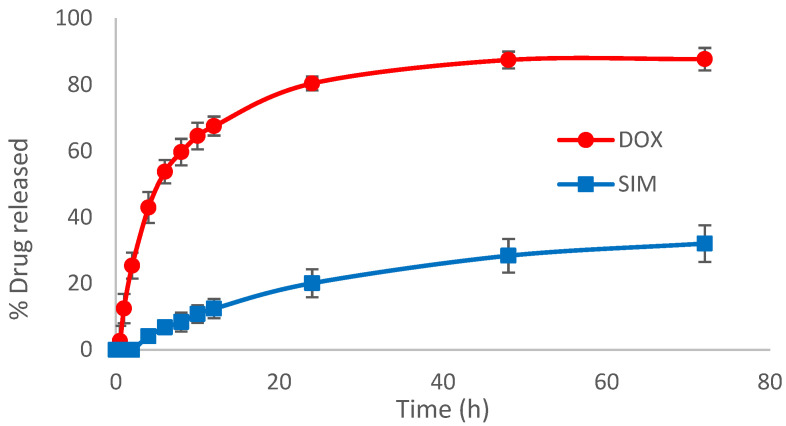
In vitro drug release profile of the optimal formulation. The concentration of the active substances in the release media were determined using the HPLC method.

The release profile of liposomes is equally accountable for achieving a favorable outcome in cancer therapy, along with the entrapped drug concentration, particle size, PdI or zeta potential, the main objective being to prevent drug leakage during blood circulation time and favor the release at the tumor site [41,42]. As previously demonstrated, different formulation factors/release conditions can influence the total percentage of the released drug [33,43]. Therefore, phosphate buffer at pH 5 was used for the release study of SIM-DOX-LCL formulation to mimic the tumor microenvironment conditions, while ethanol was used to assure sink conditions for SIM release, given that SIM is a lipophilic API [14]. In these circumstances, the release profile of the optimal formulation of SIM-DOX-LCL (Figure 6) was evidenced to be similar to what was already reported [14], DOX presenting a burst release in the first 10 to 12 h followed by a slow release, while SIM presented a slow and prolonged release for the entire duration of the study. The total percentage of the released DOX was 87.68%, while that of SIM was 32.05%. This result can be attributed to three factors: (i) the role that SIM concentration plays in the reduction in phase transition temperature (T_m_) of DPPC, (ii) to the use of a higher concentration of cholesterol and PL, and (iii) to the temperature used for the release study [34,44,45,46].

According to the literature, DOX sulfate has reduced solubility in aqueous media [37], but Rusell et al. demonstrated that its solubility is actually highly dependent on the type of the aqueous medium (buffer, serum), pH and temperature [45]. The underlying mechanism of the formation of DOX sulfate inside the liposomes involves the reaction between DOX in a neutral form and AS, a process which is reversible [37]. The study of Rusell et al. demonstrated that after increasing the temperature from 4 °C to 37 °C, the dissolution rate constant of DOX sulfate increases three times. The same study also highlighted that the dissolution rate of DOX sulfate inside the liposomes is faster than the transportation rate of DOX across the lipid bilayer [45]. Furthermore, DOX transportation from the aqueous core of liposomes into the release medium is dependent on the flip-flop movements of PL. It was thus demonstrated via molecular dynamic simulations that the addition of cholesterol in the liposomal formulation reduces the motility of the PL due to an increase in the liposomal membrane rigidity [46,47]. In view of this, we assume that during in vitro release study, both processes, namely the transportation of DOX across the liposomes’ membrane and the dissolution of DOX sulfate, are equally accountable for the extended release of DOX in a high percentage. In addition, the release of DOX from liposomes can also be positively influenced by the use of high levels of SIM during the preparation process. More precisely, Sariisik et al. demonstrated that the T_m_ of DPPC decreases when high concentrations of SIM are used, and liposomes are converted from a rigid and stable form to a more fluid and unstable form [34], which can promote the drug release from the aqueous core of liposomes at the same temperature. A similar result to ours was also reported by Li et al. [48]. Despite this, the release of SIM from liposomes was evidenced to not to be affected by these factors; it was constant regardless of the pH or PL concentration [14,49]. This phenomenon can be explained by the fact that lipophilic statins form strong bonds with PL [50], leading to a prolonged release of SIM from the liposomes’ bilayer.

In addition, it can be inferred from Table 5 that the release kinetic of DOX can be described via zero-order, Korsmeyer–Peppas, Makoi–Banakar and Weibull models (R^2^ > 0.95). This result highlights that DOX is released slowly from the optimal formulation as a result of the slow dissolution process of DOX sulfate crystals and the diffusion of DOX molecules through the lipid bilayer (the n value of Korsmeyer–Peppas kinetic model was 0.22) [51,52]. On the other hand, the release profile of SIM was better fitted with Higuchi, Korsmeyer–Peppas, Baker–Lonsdale, Makoi–Banakar and Weibull release kinetic models (R^2^ > 0.95). This suggests that SIM, like DOX, is released slowly from the optimal formulation, but in this case, the drug release does not follow the Fick’s diffusion laws (the n value for Korsmeyer–Peppas kinetic model was 0.46). More precisely, the release of SIM is caused to the same extent by the diffusion of SIM molecules through the lipid bilayer and by the “erosion” or “disintegration” of the liposomes’ membrane [51,53].

#### 2.2.2. A Comparison of the HPLC and Chronoamperometric Method Concerning the Assay of SIM and DOX from the Release Medium

Despite the promising results that have been obtained in recent decades regarding the use of liposomes in cancer therapy, there is still some controversy around this method [54]. In this respect, it has been postulated that the difference in the therapeutic response could be the consequence of a different pharmacokinetic profile of liposomes compared to free drug, which will ultimately influence the therapeutic outcome and the administered dose [55].

As a means to better understand the pharmacokinetic profile of drugs/nanoparticles while providing accessible devices to scientists and patients to quantify different compounds from simple/complex matrices rapidly and in an easy manner, the electrochemical analysis contributes to this field with a wide variety of sensors compatible with portable devices, i.e., mobile phones, with applications in research, industry or even medical units [56,57,58].

**Figure 7:**
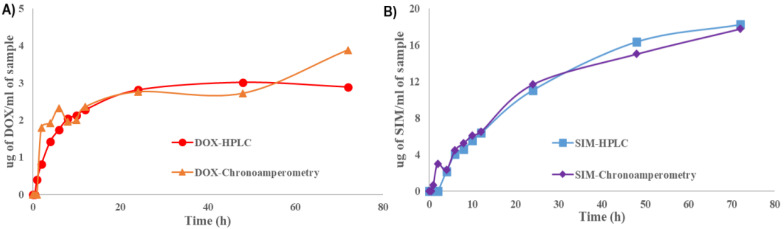
Comparison of the two analytical techniques for the release of DOX (**A**) and SIM (**B**) from the optimal formulation.

Taking into account the applicability and advantages of the electrochemical techniques, we have used a chronoamperometric method developed and optimized by Rus et al. [59] to simultaneously quantify SIM and DOX released from the optimal formulation. This novel analytical technique was assessed for its prospective implementation in the analysis of liposomes and characterization of the pharmacokinetic profile of SIM-DOX-LCL. Results have shown that the determined concentrations are similar when both techniques were used, namely HPLC and chronoamperometry (Figure 7). In the case of SIM assessment, the results are very close, while in the case of DOX, a few differences were noted at some specific time points. We assume that these variations were determined by the small concentrations of DOX that can be found in the release medium in the first hours of the study.

**Figure 8:**
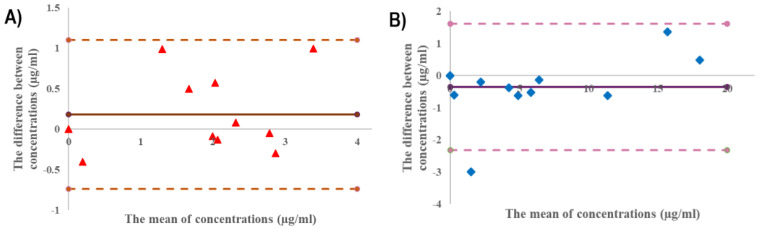
Bland–Altman graphics for the comparison of the two analytical techniques for the release of DOX (**A**) and SIM (**B**).

To obtain a detailed understanding of these results, the Bland–Altman plot was used to evaluate the agreement between the two analytical techniques [60]. The HPLC technique was considered to be the reference method. To draw up the plots, the mean concentrations for each API and the limits of agreement were calculated. Therefore, the mean of DOX concentrations was 0.18 µg/mL with the limits of agreement between 1.1 and −0.74 µg/mL, while the mean of SIM concentrations was −0.35 µg/mL with the limits of agreement between 1.62 and −2.32 µg/mL. Results (Figure 8) have evidenced that the differences between the two analytical techniques for both APIs are located between the limits of agreement, underlining that the chronoamperometry method developed by Rus et al. [59] can be used for the simultaneous assessment of SIM and DOX. This conclusion is also supported by the *p* value of the paired t-test, which was 0.13 for DOX and 0.49 for SIM, suggesting that there is no significant difference in results when the two analytical techniques were compared.

### 2.3. Stability Study

Investigating the stability of liposomes is considered to be a critical issue in the pharmaceutical development process because the intended effect depends on maintaining the liposomes’ CQAs in the target ranges during storage [30,61,62]. Previously, it was evidenced that the stability of liposomal formulations is dependent on factors such as the type of PL, cholesterol concentration, loading method of the API, temperature or pH [61,63,64,65]. The ICH guideline pointed out that “a 5% change in assay from its initial value” is considered significant, and additional tests are required [66]. The stability of SIM-DOX-LCL optimal formulation was evaluated in terms of liposomal size, PdI, zeta potential and the percentage of drug retained over four weeks. The storage conditions were established by considering the specifications mentioned in the summary of product characteristics of Doxil^®^ and Caelyx [67,68], two liposomal products with DOX approved by the regulatory agencies.

**Figure 9:**
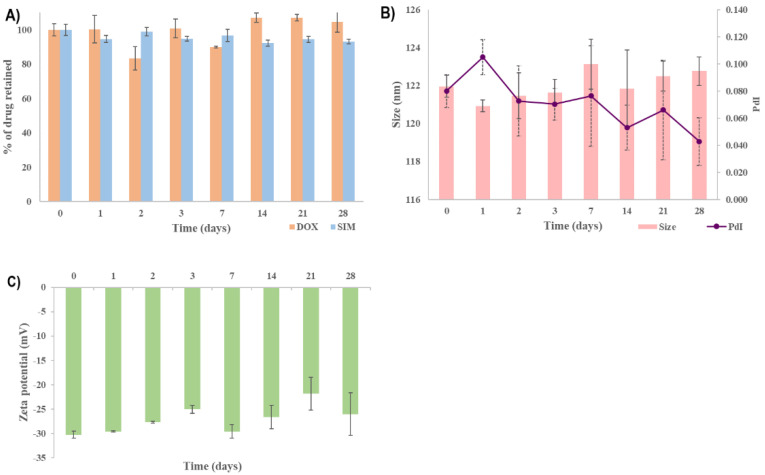
The stability of SIM-DOX-LCL optimal formulation: The % of drug retained (**A**); liposomal size and PdI (**B**); zeta potential (**C**).

Under these experimental conditions, the SIM-DOX-LCL optimal formulation presented good stability over the tested period. The % of DOX retained (Figure 9A) was constant for the entire duration of the study, while the % of SIM retained decreased to 93% after four weeks. Evidence has shown that the use of PL with a high chain length and T_m_ produces liposomes with increased stability, while the use of a low temperature (<T_m_) to store the liposomal formulations minimizes lipid mobility, thus preventing drug leakage and liposomal coalescence [63,64]. Additionally, the encapsulation of DOX using the AS gradient determines the formation of DOX-sulfate precipitate with a very low permeability through the liposomal membrane, thereby ensuring efficient retention of the drug inside the liposomes for a long period of time [32,69]. On the other hand, the stability of SIM in aqueous mediums was evidenced to be highly affected by factors such as the composition of the aqueous medium, pH or temperature [70,71]. Because the molecular structure of SIM includes a lactone moiety, SIM is highly susceptible to degradation to an acidic form [70]. Previous studies evidenced that the half-life of SIM in water at room temperature is 12.9 days [72], and an acidic pH and a lower temperature can delay the conversion [70]. Therefore, we assume that the low temperature used when storing the optimized SIM-DOX-LCL formulation prevented SIM degradation.

The other referenced CQAs, namely particle size, PdI and zeta potential, were preserved in the previously established target ranges, as can be seen in Figure 9B,C. In light of this, it can be concluded that the increased stability of SIM-DOX-LCL optimal formulation is ascribed equally to the liposomal formulation, storage conditions and to the active loading method of DOX.

### 2.4. Cell Proliferation Assay

It is a fact that breast and lung cancer present the highest mortality among oncologic patients and, consequently, many studies are focusing on finding treatment options that can inhibit tumor progression [6,10,26]. Usually, the therapeutic schemes for breast and lung cancer involve drug combinations that can target different intratumoral/intracellular pathways to inhibit tumor progression and avoid drug resistance [6,10]. The most recent results obtained from clinical trials have suggested that statins combined with chemotherapeutic agents have a negative influence on the progression of both lung and breast cancer [6,73]. The results obtained in our study also indicate that SIM and DOX present a synergistic effect on both T47D-Kbluc human mammary ductal carcinoma cell line and A549 human pulmonary cancer cell line when the two active substances are combined in a free form. In this case, the cell viability was less than 50% for all drug combinations, making it difficult to estimate the IC_50_ (Table 6).

The long circulating liposomes loaded with DOX (DOX-LCL) were observed to increase the IC_50_ of DOX on both cell lines, possibly due to the prolonged drug release [14,74]. In the case of SIM-LCL formulation, a two-fold decrease in the IC_50_ of SIM on T47D-KBluc cell line was noted. On the other hand, the results obtained on the A549 cell line highlighted that there is only a small difference in the IC_50_ value of SIM-LCL and free SIM. It should be mentioned that empty liposomes (E-LCL) were assessed in conjunction with the treatments to exclude their influence on cell viability. The results highlighted that cell viability for E-LCL was 99% (data not presented), and all the reported results exclusively represent the influence of the treatments.

**Figure 10:**
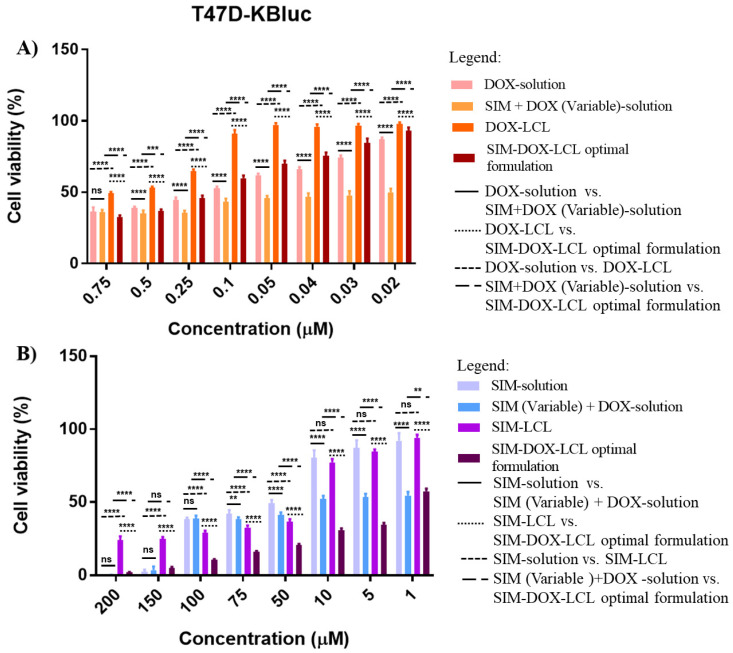
The effects of DOX (**A**) and SIM (**B**) treatments as monotherapy or in combination, on the proliferation of T47D-KBluc human mammary ductal carcinoma cell line. ns: not significant (*p*-value > 0.05); **: *p*-value < 0.01; ***: *p*-value < 0.001; ****: *p*-value < 0.0001.

Apart from these results, SIM-DOX-LCL optimal formulation also documented increased resistance in the T47D-KBluc human mammary ductal carcinoma cell line when low concentrations of SIM (7.5–0.2 µM) and DOX (0.75–0.02 µM) were used (Figure 10A), the DOX IC_50_ value increasing 1.5 times for liposomes compared to free DOX. This result is in agreement with another study, which demonstrated that the T47D-KBluc cell line is more resistant to SIM therapy than other breast cancer cell lines, and higher concentrations are required to achieve an antiproliferative effect [75]. Moreover, the same study evidenced that SIM, atorvastatin and rosuvastatin can increase the expression of HMGCoA reductase genes in some breast cancer cell lines, therefore inducing drug resistance [75]. By comparing the results obtained with what was already reported, it can be observed that the use of higher concentrations of SIM (1–200 µM) and DOX (0.1–20 µM) in the liposomal formulation decrease the SIM IC_50_ by 30 times compared to free SIM, and 16 times compared to SIM-LCL.

**Figure 11:**
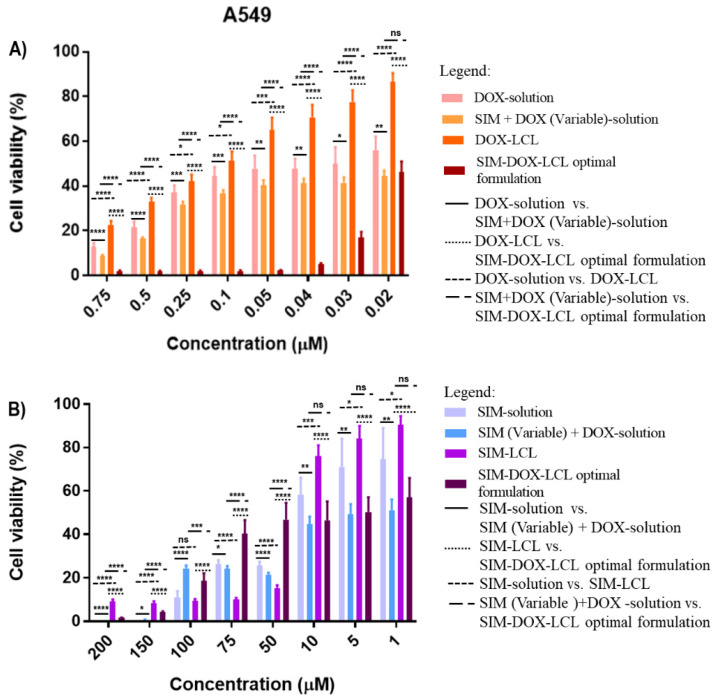
The effects of DOX (**A**) and SIM (**B**) treatments as monotherapy or in combination, on the proliferation of A549 human pulmonary cancer cell line. ns: not significant (*p*-value > 0.05); *: *p*-value < 0.05; **: *p*-value < 0.01; ***: *p*-value < 0.001; ****: *p*-value < 0.0001.

In contrast with the previous results, the SIM-DOX-LCL optimal formulation highlighted a higher level of activity against the A549 human pulmonary cancer cell line when smaller concentrations of SIM (7.5–0.2 µM) and DOX (0.75–0.02 µM) were used (Figure 11A). The IC_50_ value of DOX decreased 2.5 times compared to free DOX, and 6.8 times compared with DOX-LCL. In this respect, Aksoy and Ceylan demonstrated that statins have a different impact on A549 human pulmonary cancer cell line. More precisely, they reported that high concentrations of SIM determine an increase in cholesterol concentration in the cell membrane, with a direct impact on its fluidity and permeability [76]. Bearing this in mind, we assume that higher concentrations of the SIM-DOX-LCL optimal formulation determined an increase in cholesterol content of cell membrane, which further limited the internalization of liposomes, thus inducing the resistance to treatment (Figure 11B) and a 1.17 times increase in SIM IC_50_ value compared to free SIM or SIM-LCL.

The results obtained in this study suggest that SIM enhances the anticancer properties of DOX in a free form and co-encapsulated in liposomes, achieving a synergistic effect on both studied human cancer cell lines. However, it must be taken into consideration that the antiproliferative potential of SIM-DOX-LCL is concentration dependent, and a thorough analysis should be performed before establishing the dosing regimens for the future in vivo studies. In addition to these, additional studies are required to establish the pharmacokinetic profile of SIM-DOX-LCL optimal formulation as a means to ensure the desired drug ratio at the tumor site.

## 3. Material and Methods

### 3.1. Materials

Doxorubicin hydrochloride (DOX) from Merck KGaA (Darmstadt, Germany), simvastatin (SIM) from Biocon Limited (Bengaluru, India), 1,2-dipalmitoyl-sn-glycero-3-phosphocholine (DPPC) and N-(carbonyl-methoxypolyethylenglycol-2000)-1,2-distearoylsn-glycero-3-phosphoethanolamine (Na-salt; MPEG-2000-DSPE), from Lipoid GmbH (Ludwigshafen, Germany); cholesterol (CHO) from sheep wool, resazurin and fetal bovine serum, from Merck KGaA (Darmstadt, Germany); sodium chloride and ammonium sulfate (AS), from Chemical Company (Iasi, Romania). For cell culture assay, Dulbecco’s modified Eagle medium (DMEM) and phosphate-buffered saline (PBS) were purchased from Gibco (Paisley, UK), RPMI 1640 medium with GlutaMAX^TM,^ from ThermoFisher Scientific (Waltham, MA, USA); resazurin and fetal bovine serum (FBS) were from Sigma Aldrich (Steinheim, Germany).

All the other solvents and reagents were of analytic grade purity, and were commercially available.

### 3.2. Methods

#### 3.2.1. The Optimization Process of SIM-DOX-LCL

According to the ICH guideline for pharmaceutical development Q8(R2), the implementation of the QbD approach in the development of pharmaceutical formulations/products has to follow a specific number of steps, which include defining the QTPP, identifying the CQAs, interconnecting the material attributes/process parameters with the CQAs, and defining the design space [18]. The first steps of the QbD approach were successfully completed in the previous study published by Barbalata et al. [14], where the critical material attributes (CMAs) and critical process parameters (CPPs) were identified through a screening experimental study. In the current study, the SIM-DOX-LCL formulation was optimized, and the design space was defined by considering previously obtained results. Thereby, a secondary risk analysis using the FMEA was performed at first to reduce the number of factors that need to be optimized. The factors that were formerly identified as critical for the SIM-DOX-LCL CQAs, namely the concentration of PL, the concentrations of DOX and SIM and the pH of the AS solution [14], were re-evaluated considering the severity of their produced effects (S) on the CQAs of the SIM-DOX-LCL formulation, the occurrence (O) and how difficult it was to detect the source of the problem (D). For each CMA and CPP, a RPN was calculated as follows:

RPN = S × O × D (2)

The factors that counted the highest RPN were included in a DoE conceived in Modde 13 software (Sartorius Stedim Data Analytics AB, Umea, Sweden). The CQAs that constituted the matrix of dependent variables were the entrapped drug concentration, EE%, liposomal size, PdI and zeta potential. The DoE was a central composite face design, quadratic model, with 17 experiments of which three were replicates. Modde 13 software was also used for the statistical analysis of the data and to define the design space. The optimal formulation was prepared in triplicate, the results being expressed as average ± standard deviation. For each response, the experimental value was compared to the predicted value, and the residual value was calculated using the following formula:

(3) $$\mathrm{Residual}\;\mathrm{value}\;=\frac{\mathrm{Predicted}\;\mathrm{results}-\mathrm{Observed}\;\mathrm{results}}{\mathrm{Predicted}\;\mathrm{results}}\times 100\;$$

#### 3.2.2. Preparation of Liposomes

##### Preparation of Liposomes Loaded with SIM

SIM-LCL were prepared using a modified method of the lipid film hydration technique reported by Porfire et al. [77]. In this respect, lipidic components, namely DPPC, MPEG-2000-DSPE, CHO (molar ratio 95:5:10) and SIM (different concentrations according to the DoE) were dissolved in ethanol in a round-bottom flask, after which the solvent was evaporated under a vacuum. The obtained lipid film was hydrated with an AS solution (pH = 5.0) using the rotavapor. The obtained liposomal suspension was downsized using LiposoFast LF-50 equipment (Avestin Europe GmbH, Germany) and polycarbonate membranes. The achievement of liposomes of the desired size was made possible by passing the liposomal suspension three times through the membrane with the diameter pore size of 800 nm, and seven times through each of the membranes with the diameter pore size of 200 and 100 nm. The final liposomal suspension was purified against saline using a Slide-A-Layzer cassette with a molecular cut-off of 10 kDa for 24 h at 4 °C.

##### Preparation of Liposomes Loaded with SIM and DOX

The encapsulation of DOX into SIM-LCL was performed using a modified process of the active loading method with AS [43,78,79]. Therefore, the SIM-LCL formulation obtained after the extrusion process was dialyzed against saline for three hours at room temperature to establish a pH gradient. Subsequently, DOX was encapsulated into liposomes by incubating SIM-LCL with DOX (different concentrations according to the DoE) in a volume ratio of 4 to 1 at 60 °C for 15 min using the rotavapor. The SIM-DOX-LCL formulation was purified via dialysis against saline for 24 h at 4 °C.

##### Preparation of Liposomes Loaded with DOX

The preparation of DOX-LCL was performed following the techniques described at the preparation of SIM-LCL and SIM-DOX-LCL, but without the addition of SIM in the lipid bilayer.

##### Preparation of Empty Liposomes

The preparation of E-LCL was performed following the technique described at the preparation of SIM-LCL, but without the addition of SIM in the lipid bilayer.

#### 3.2.3. Characterization of Liposomes

##### Determination of SIM and DOX Entrapped Concentration. Determination of EE%

The SIM and DOX entrapped concentrations were determined using an HPLC method developed and validated by Barbalata et al. [14]. Firstly, the liposomes were diluted in methanol in a volume ratio of 1 to 50, and the stock solution was analyzed using an Agilent 1100 Series HPLC system (Agilent Technologies, Santa Clara, CA, USA) equipped with a Zorbax C18 column (3.5 µ) (Phenomenex, Torrance, CA, USA). Since SIM and DOX have different physiochemical properties, the chromatographic method involved a gradient elution with acetonitrile and formic acid 0.1% as mobile phases, while the absorbance was read at 245 nm. In these conditions, the retention time of DOX was 0.95 min, while that of SIM was 4.5 min.

The EE% for SIM and DOX was determined using the formula:

(4) $$\mathrm{EE}\%=\frac{\mathrm{Encapsulated}\;\mathrm{drug}\;\mathrm{concentration}}{\mathrm{Total}\;\mathrm{drug}\;\mathrm{concentration}}\times 100$$

##### Determination of Liposomal Size, PdI and Zeta Potential

For the measurement of the liposomal size and PdI, the dynamic light scattering technique was used, while for zeta potential, the laser Doppler electrophoresis was applied. All the measurements were performed in triplicate at room temperature with a Zetasizer Nano ZS90 analyzer (Malvern Instruments Co., Malvern, UK) after diluting the samples with double distilled water (1:100 *v*/*v*). The results were expressed as an average.

##### In Vitro Release Study

The in vitro release study was performed for the optimal formulation, in triplicate. A volume of 2 mL of SIM-DOX-LCL formulation was placed in a dialysis bag, and 100 mL of phosphate buffer pH 5.0 with 30% ethanol was used as release medium. The release study was conducted at 37 ± 0.5 °C on continuous stirring (100 rpm) for 72 h [14,49]. At predefined intervals, a sample of 3 mL was withdrawn and replaced with fresh medium. The samples were analyzed using two different analytical techniques, namely HPLC and chronoamperometry. The results obtained from the two analytical techniques were compared using a paired t-test and a Bland–Altman graphic.

The HPLC analysis was performed according to the previously mentioned method, while chronoamperometry analysis was carried out via a method reported by Rus et al. [59] and adapted for this study. Briefly, the analytical parameters such as dynamic range, the oxidation potential applied for each analyte (0.5 V for DOX and 0.95 V for SIM) and the correlation between the current intensity and the concentration of DOX and SIM expressed through calibration curves were kept at the levels reported in the mentioned study. An adjustment of the ethanol content of the samples was necessary, since the analytical parameters for chronoamperometry were determined by using 25% ethanol. This sample dilution with phosphate buffer (from 30% ethanol to 25% ethanol) was considered in the calculation of the DOX and SIM content, respectively. The chronoamperometry tests were performed on screen-printed electrodes with a working electrode of 4 mm diameter based on carbon, a Ag/AgCl pseudo-reference electrode and carbon based auxiliary electrode (Metrohm DropSens; Oviedo, Spain), using a multi-channel potentiostat/galvanostat Autolab MAC80100 (Metrohm, Utrecht, The Netherlands).

##### Stability Study

The stability study was performed on the optimal SIM-DOX-LCL formulation for 28 days. The liposomes were kept at 4–8 °C and at predefined periods, samples were withdrawn and analyzed in triplicate with respect to the entrapped drug concentration (expressed as % of drug retained), EE%, liposomal size, PdI and zeta potential.

The % of drug retained was calculated using the following equation:

(5) $$\%\;\mathrm{of}\;\mathrm{drug}\;\mathrm{retained}=\frac{\mathrm{the}\;\mathrm{entrapped}\;\mathrm{drug}\;\mathrm{concentration}\;\mathrm{on}\;\mathrm{day}\;n}{\mathrm{the}\;\mathrm{entrapped}\;\mathrm{drug}\;\mathrm{concentration}\;\mathrm{on}\;\mathrm{day}\;0}\times 100$$

The results were expressed as average ± standard deviation.

#### 3.2.4. Cell Culture

In this study, two cancerous cell lines were used: T47D-KBluc human mammary ductal carcinoma cell line and A549 human pulmonary cancer cell line (American Type Culture Collection, Manassas, VA, USA). Cells were cultured in the recommended medium containing 10% inactive FBS and were kept at 37 °C in a 5% CO_2_ humidified atmosphere. The medium was changed every 2–3 days and the cells were subcultured once they reached 70–80% confluence.

#### 3.2.5. Cell Proliferation Assay

Prior to any treatment exposure, cells were seeded in 96-well plates (6 × 10^4^ cells/mL for A549 cell line and 2 × 10^5^ cells/mL for T47D-KBluc cell line) and were left for 24 h to attach to the plate. Subsequently, the cell lines were exposed to different treatments for 48 h, as shown in Table 7.

Cell viability has been assessed using a resazurin-based test. For this purpose, cells have been incubated with a resazurin solution of 200 µM for 4 h at 37 °C in a 5% CO_2_ humidified atmosphere, and the fluorescence (λ_excitation_ = 530–525; λ_emission_ = 590–535) was read using a Synergy 2 Multi-Mode Microplate Reader. The experiments were performed in triplicate. The results were expressed as % of cell viability compared to the control.

#### 3.2.6. Statistical Analysis

The statistical analysis of the DoE was performed using Modde 13 software (Sartorius Stedim Data Analytics AB, Umea, Sweden), while the data that resulted from the antiproliferative studies was analyzed using SigmaPlot 11 software (Inpixon GmbH, Dusseldorf, Germany). The ANOVA test was used to evaluate the differences between the investigated factors and the obtained results at 95% confidence level. The release profile of SIM and DOX were fitted through multiple kinetic models using DDSolver add-ins software.

## 4. Conclusions

The process knowledge gained from the screening and optimization study of SIM-DOX-LCL contributed to the development of an optimal formulation that meets the QTPP, which was defined in conformity with the scientific literature and international guidelines. Through this study, it was evidenced that the most critical factors responsible for achieving the quality profile of SIM-DOX-LCL are PL, SIM and DOX concentration.

The in vitro characterization of the optimal formulation (cytotoxicity study, drug release study) recommends SIM-DOX-LCL for future in vivo animal studies, since the drug combination presents a synergistic effect on both T47D-KBluc human mammary ductal carcinoma cell line and A549 human pulmonary cancer cell line, and the liposomal formulation presents a prolonged drug release. However, special consideration should be given to the administered dose of the liposomal formulation, since different concentrations of the same formulations had an opposite antiproliferative effect on the tested cancer cell lines. The applied chronoamperometric method for the simultaneous quantification of SIM and DOX evidenced the ability to quantify both APIs at low concentration levels in the release medium, but additional optimization studies are required to increase its selectivity before using complex matrices for therapeutic drug monitoring in animal models and pharmacokinetic studies.

Taking these positive results into consideration, the research group intends to carry out additional studies concerning the pharmacokinetic profile, antitumor efficacy or toxicity profile, to establish the in vivo benefits of associating SIM and DOX in a liposomal formulation.

## Figures and Tables

**Table 1:** The formulations and the experimental results of the optimization DoE.

Exp Name	X_1_	X_2_	X_3_	Y_1_	Y_2_	Y_3_	Y_4_	Y_5_	Y_6_	Y_7_
N1	44	0.25	2	246.97	101.22	29.17	48.66	125.36	0.03	−31.96
N2	77	0.25	2	186.59	106.21	22.49	51.06	129.80	0.06	−30.23
N3	44	0.75	2	398.54	415.56	45.77	98.28	122.73	0.04	−36.20
N4	77	0.75	2	413.58	329.63	45.75	99.95	169.40	0.04	−40.16
N5	44	0.25	12	1193.98	92.90	23.84	75.13	105.50	0.07	−34.03
N6	77	0.25	12	1507.69	117.29	29.79	56.38	117.00	0.05	−31.13
N7	44	0.75	12	1642.61	316.88	32.69	96.86	114.60	0.06	−35.20
N8	77	0.75	12	2454.31	386.73	48.94	76.71	115.03	0.06	−36.43
N9	44	0.5	7	902.89	183.50	30.94	84.16	117.36	0.04	−29.00
N10	77	0.5	7	1328.51	197.13	44.84	86.54	118.90	0.02	−46.30
N11	60.5	0.25	7	593.71	100.66	20.33	91.30	119.66	0.03	−39.46
N12	60.5	0.75	7	738.08	366.77	25.10	72.70	120.43	0.05	−34.86
N13	60.5	0.5	2	358.02	223.18	43.80	98.36	124.76	0.03	−37.53
N14	60.5	0.5	12	603.94	106.21	12.07	91.90	109.20	0.08	−37.26
N15	60.5	0.5	7	1220.93	199.90	40.60	94.77	121.30	0.04	−36.43
N16	60.5	0.5	7	1220.93	225.40	42.17	98.38	114.50	0.04	−33.80
N17	60.5	0.5	7	1308.64	188.26	44.65	86.34	117.33	0.04	−33.33

**X_1_:** PL concentration (mM); **X_2_**: DOX concentration (mM); **X_3_**: SIM concentration (mM); **Y_1_**: SIM entrapped concentration (µg/mL); **Y_2_**: DOX entrapped concentration (µg/mL); **Y_3_**: SIM EE% (%); **Y_4_**: DOX EE% (%); **Y_5_**: Liposomal size (nm); **Y_6_**: Liposomal PdI; **Y_7_**: Zeta potential (mV).

**Table 2:** The statistical parameters of the DoE.

Response	Summary of Fit				ANOVA	
	R^2^	Q^2^	Model Validity	Reproducibility	Regression Model	Lack of Fit
Y_1_	0.988	0.954	0.534	0.994	0.000 *	0.156
Y_2_	0.993	0.922	0.947	0.968	0.000 *	0.812
Y_3_	0.976	0.874	0.841	0.955	0.000 *	0.53
Y_4_	0.974	0.851	0.974	0.896	0.000 *	0.905
Y_5_	0.95	0.898	0.999	0.69	0.000 *	0.997
Y_6_	0.975	0.812	0.782	0.966	0.000 *	0.419
Y_7_	0.932	0.741	0.947	0.729	0.011	0.812

**Y_1_**: SIM entrapped concentration (µg/mL); **Y_2_**: DOX entrapped concentration (µg/mL); **Y_3_**: SIM EE% (%); **Y_4_**: DOX EE% (%); **Y_5_**: Liposomal size (nm); **Y_6_**: Liposomal PdI; **Y_7_**: Zeta potential (mV); **R^2^**: The model fit; **Q^2^**: The prediction precision. * *p*-value < 0.001

**Table 3:** The regression coefficients.

		b_0_	b_1_	b_2_	b_3_	b_11_	b_22_	b_33_	b_12_	b_13_	b_23_
Y_1_	Coefficient value	1172.670	200.460	292.833	652.068	-	−216.225	-	-	208.712	189.455
	*p*-value	0.000 *	0.002	0.000 *	0.000 *	-	0.019	-	-	0.003	0.004
Y_2_	Coefficient value	204.523	-	140.534	−18.360	-	29.190	-	−19.189	35.404	-
	*p*-value	0.000 *	-	0.000 *	0.041	-	0.032	-	0.037	0.003	-
Y_3_	Coefficient value	41.857	3.301	10.077	−2.202	-	−9.919	-	-	5.016	-
	*p*-value	0.000 *	0.006	0.000 *	0.041	-	0.001	-	-	0.001	-
Y_4_	Coefficient value	94.219	−9.023	12.735	-	-	−18.979	-	-	−10.208	−11.893
	*p*-value	0.000	0.002	0.000	-	-	0.000 *	-	-	0.001	0.001
Y_5_	Coefficient value	117.621	-	-	−7.305	-	-	-	−2.842	-	-
	*p*-value	0.000 *	-	-	0.000 *	-	-	-	0.018	-	-
Y_6_	Coefficient value	0.039	−0.007	0.011	0.022	−0.015	-	0.013	0.005	-	−0.012
	*p*-value	0.000 *	0.001	0.001	0.000 *	0.000	-	0.001	0.016	-	0.001
Y_7_	Coefficient value	−34.520	-	−3.099	-	4.931	-	-	−1.747	-	-
	*p*-value	0.000 *	-	0.002	-	0.026	-	-	0.020	-	-

**Y_1_**: SIM entrapped concentration (µg/mL); **Y_2_**: DOX entrapped concentration (µg/mL); **Y_3_**: SIM EE% (%); **Y_4_**: DOX EE% (%); **Y_5_**: Liposomal size (nm); **Y_6_**: Liposomal PdI; **Y_7_**: Zeta potential (mV); **b_0_**: model constant; **b_1_, b_2_, b_3_**: linear coefficients; **b_11_, b_22_, b_33_**: quadratic coefficients; **b_12_, b_13_, b_23_**: interaction coefficients between the three factors. * *p*-value < 0.001.

**Table 4:** The composition of the optimal formulation and its actual physicochemical characteristics vs. predicted ones.

Formulation Factors		Responses					
Factor	Value (mM)	Responses	Target	Predicted Value	Experimental Value	SD	Residual Value (%)
X_1_	56	Y_1_ (µg/mL)	>1200	2089.32	2096.03	129.08	−0.32
		Y_2_ (µg/mL)	>120	313.31	273.76	38.40	12.62
X_2_	0.68	Y_3_ (%)	>40	43.53	41.95	2.48	3.63
		Y_4_ (%)	>50	83.30	76.56	3.08	8.09
X_3_	12	Y_5_ (nm)	<200	113.00	116.93	4.38	−3.48
		Y_6_	<0.2	0.074	0.055	0.022	25.68
		Y_7_ (mV)	<−30	−41.31	−31.70	1.45	23.26

**SD**: Standard deviation; **X_1_**: PL concentration; **X_2_**: DOX concentration; **X_3_**: SIM concentration; **Y_1_**: SIM entrapped concentration; **Y_2_**: DOX entrapped concentration; **Y_3_**: SIM EE%; **Y_4_**: DOX EE%; **Y_5_**: Liposomal size; **Y_6_**: Liposomal PdI; **Y_7_**: Zeta potential.

**Table 5:** The correlation coefficient (R^2^) of SIM and DOX when different release kinetic models were applied.

Kinetic Model	R^2^	
	DOX	SIM
Zero-order with Tlag	0.954	0.899
First-order with Tlag	0.947	0.928
Higuchi with Tlag	0.731	0.995
Korsmeyer–Peppas with Tlag	0.972	0.995
Hixson–Crowell with Tlag	0.866	0.918
Hopfenberg with Tlag	0.947	0.927
Baker–Lonsdale with Tlag	0.938	0.996
Makoid–Banakar with Tlag	0.996	0.996
Weibull with Tlag	0.972	0.975

**Table 6:** The IC_50_ values of SIM and DOX.

Pharmaceutical Formulation	Concentration (µM)		IC_50_ (µM)	
	SIM	DOXO	T47D	A549
SIM-solution	1–200	-	55.88	18.26
SIM-LCL		-	29.02	18.74
SIM (Variable) + DOX-solution		IC_50_ value	N.A.	N.A.
DOX-solution	-	0.02–0.75	0.11	0.045
DOX-LCL	-		0.75	0.124
SIM+ DOX (Variable)-solution	IC_50_ value		N.A.	N.A.
SIM-DOX-LCL optimal formulation (SIM:DOX = 10:1 molar ratio)	1–200	0.1–20	1.81	21.44
	0.2–7.5	0.02–0.75	0.17	0.018

N.A.: Not applicable.

**Table 7:** The pharmaceutical formulations and the concentrations used for SIM and DOX in the antiproliferative studies.

Treatment	Concentration (µM)	
	SIM	DOXO
SIM-solution	1–200	-
SIM-LCL		-
SIM (variable) + DOX-solution		IC_50_ value
DOX solution	-	
DOX-LCL	-	0.02–0.75
SIM + DOX (variable)-solution	IC_50_ value	
SIM-DOX-LCL optimal formulation (SIM:DOX = 10:1 molar ratio)	1–200	0.1–20
	0.2–7.5	0.02–0.75

## Data Availability

Data is contained within the article.
